# Optimizing Treatment Outcomes in Crohn’s Disease: A Comprehensive Systematic Review and Meta-Analysis of Regenerative Therapies with Emphasis on Platelet-Rich Plasma

**DOI:** 10.3390/ph17111519

**Published:** 2024-11-12

**Authors:** Marcia Carolina Mazzaro, Ana Emília Carvalho de Paula, Livia Bitencourt Pascoal, Livia Moreira Genaro, Isabela Machado Pereira, Bruno Lima Rodrigues, Priscilla de Sene Portel Oliveira, Raquel Franco Leal

**Affiliations:** 1 Inflammatory Bowel Disease Research Laboratory, Gastrocenter, Colorectal Surgery Unit, School of Medical Sciences, University of Campinas (Unicamp), Campinas 13083-878, Brazil; 2 Healthy Sciences Institute, Federal University of Jataí (UFJ), Jataí 75804-615, Brazil

**Keywords:** Crohn’s disease, platelet-rich plasma, regenerative therapy, inflammation

## Abstract

Background/Objectives: Crohn’s disease (CD) is a chronic inflammatory disorder that significantly affects patients’ quality of life; conventional treatments often provide limited relief. Methods: This systematic review and meta-analysis explored the potential of regenerative therapies, particularly platelet-rich plasma (PRP), as an adjunctive treatment for CD. The study protocol was registered with PROSPERO (CRD42024576683), and a comprehensive search was conducted across major databases, such as PubMed, EMBASE, and the Cochrane Central Register of Controlled Trials. The search included terms related to CD and PRP. Studies assessing the efficacy of PRP in CD treatment were selected. Statistical analysis was conducted using the PICO framework with R software (version 4.3.2) and meta-package. Results: Of the 29 studies identified, 10 met the inclusion criteria, comprising pilot studies and controlled trials. Nine studies focused on Crohn’s disease perianal fistulas (CDPF), and one focused on colonic CD. Among 138 patients with CDPF, 82.44% showed some fistula healing after PRP treatment, with 48.05% achieving complete resolution. In a sub-analysis, combining PRP with a stromal vascular fraction (SVF) resulted in a 58.62% complete healing rate, whereas combining PRP with adipose-derived stem cells (ASCs) showed even higher efficacy at 85.89%. PRP treatment alone resulted in a lower complete healing rate of 38.51%. PRP was well tolerated, with minor side effects such as localized pain. Conclusions: These findings suggest that PRP, especially when combined with stem cells, offers a promising new approach for treating CD. However, larger trials are needed to confirm its long-term benefits and refine its clinical applications.

## 1. Introduction

A principal subtype of inflammatory bowel disease (IBD), Crohn’s disease (CD) is a chronic autoimmune condition affecting any part of the digestive tract, particularly the terminal ileum [1]. CD is a chronic inflammatory mucosal disease that extends through all layers of the intestinal wall (transmural) and affects the intestinal wall segmentally and asymmetrically (skip lesions). This can lead to the formation of ulcerations, fistulas, strictures, and granulomas and characteristically evolves through periods of exacerbation and remission [2]. CD treatment depends on severity, location, and disease subtype. Risk factors for the aggressive form include age < 30 years at diagnosis, extensive involvement, perianal disease, deep ulcers, previous surgery, and stenosing or penetrating disease [3].

Several drugs are currently used to treat CD; therapeutic interventions aim to control symptoms, induce clinical remission, and maintain remission with minimal adverse effects [4]. Despite medical management, 70–80% of patients with CD eventually require surgery, most commonly for complications of strictures, fistulas, or abscesses [5]. Additionally, approximately 30% of patients do not respond adequately to induction therapy with Tumor Necrosis Factor (TNF) inhibitors. Approximately 40% of patients who initially show a response develop secondary failure [6]. The causes of secondary failure are nonadherence to treatment with anti-TNF agents, drug immunogenicity, or persistent inflammatory activity despite sufficient anti-TNF levels [7].

Specifically, new therapies have been proposed for treating fistulas in CD, as those used to date, both clinical and surgical or combined, are ineffective in many cases [8,9]. The use of regenerative therapies has been studied because the origin of fistulas in CD involves a sequence of changes in which an epithelial barrier defect favors the entry of pathogens into the gut mucosa. These events promote an inflammatory reaction and stimulate mechanisms that promote disordered cell growth and local overstimulation of wound healing [10].

Platelet-rich plasma (PRP), a type of regenerative therapy, is derived from the processing of peripheral blood and contains a high concentration of platelets [11]. Studies have shown that platelets participate in the inflammatory process by releasing substances that interact with endothelial cells and leukocytes to modulate the inflammatory response [12,13]. Some immunomodulatory factors, such as PDGF, TGF-β, CD40L, and CD154, have been identified among these factors. Platelets are vital not only for hemostasis, but also for the immunological and inflammatory aspects of tissue healing. They release various cytokines and chemokines, such as TGF-β, IL-1β, CD40L, CXCL7, CXCL4, CXCL4L1, CCL5, CXCL1, CXCL8, CXCL5, CXCL12, CCL2, and CCL3, which contribute to the inflammatory response, a fundamental aspect of the healing process. Furthermore, platelets express chemokine receptors, including CCR1, CCR3, CCR4, and CXCR4, allowing them to regulate inflammation [12].

Platelets can regulate local inflammation through anti-inflammatory cytokines like TGF-β, preventing excessive leukocyte recruitment. This modulation of inflammation is crucial for maintaining the balance necessary for effective tissue healing and preventing chronic inflammatory conditions [14]. The cellular composition of PRP influences its bioactive molecular profile, with platelets associated with anabolic signaling and leukocytes associated with catabolic signaling. TGF-β is the principal immunomodulatory molecule with immunosuppressive actions that influence Treg differentiation [15]. Due to this specific immunomodulatory characteristic of Treg differentiation based on TGF-β, PRP has emerged as a potential therapeutic option for specific inflammatory diseases, particularly refractory CD [16].

## 2. Results

### 2.1. Literature Search and Study Characteristics

**Figure 1:**
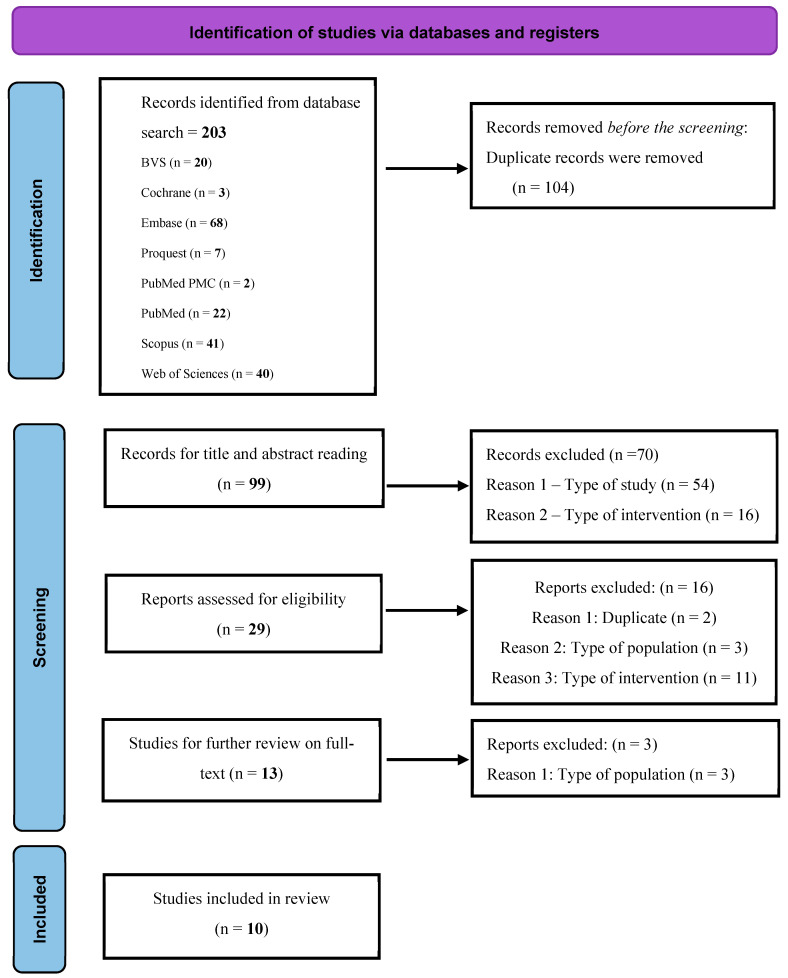
Prisma 2020 flow diagram for the new systematic review included searches of databases and registers 18.

A thorough search conducted across nine databases until 19 March 2024 retrieved 201 potentially relevant references using the Rayyan QCRI17 system [17] and Preferred Reporting Items for Systematic Reviews and Meta-Analyses (PRISMA) guidelines [18]. The library staff at the School of Medical Sciences (SMS) of the University of Campinas (Unicamp) facilitated the collection of articles and assisted in the search and identification of references. After reviewing the titles and abstracts, 104 were excluded because they were duplicates, and 97 did not meet the eligibility criteria based on our research design. Consequently, 29 studies were assessed for eligibility, 13 of which were selected for an in-depth full-text review. Of these, 10 studies met the inclusion criteria and were incorporated into this review, utilizing methodologies ranging from pilot studies to uncontrolled clinical trials [19,20,21,22,23,24,25,26,27,28]. A flow diagram of this review is shown in Figure 1. In analyzing studies focusing on CD and related conditions, this evaluation identified two prospective observational studies, two pilot studies, two prospective studies, one retrospective observational study, one prospective cohort study, one case series, and one prospective, uncontrolled, single-center study. A total of 143 patients were analyzed, of whom 138 had perianal CD (PCD) and 5 refractory colonic CD.

### 2.2. Efficacy of Platelet-Rich Plasma Injections for Treating Perianal CD

**Figure 2:**
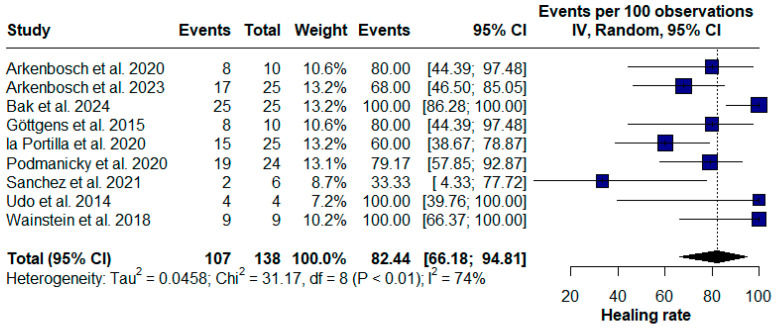
Forest plot of the overall healing rate for Crohn’s disease (CD) perianal fistulas [16,19,20,22,24,25,26,27,28].

The diverse methodological approaches and study types highlighted in these investigations underscore the complexity and potential of PRP in treating various perianal and anal fistulas, particularly CD. To evaluate the efficacy of PRP, we initially included only studies that addressed the treatment of perianal fistulas. Of the 138 patients with PCD reported across nine studies, 107 achieved some degree of fistula healing after treatment, representing an overall efficacy of 82.44% (95% confidence interval [CI] [66.18; 94.81], chi^2^ = 31.17; I^2^ = 74%) (Figure 2).

**Figure 3:**
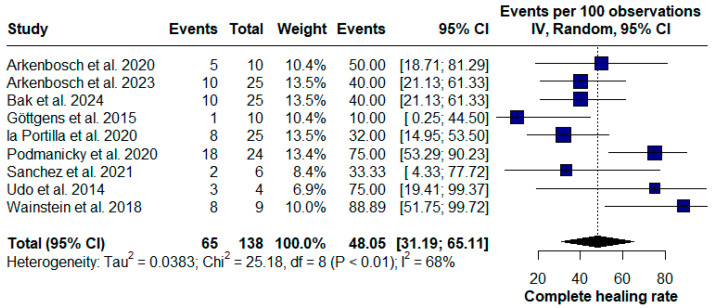
Forest plot of the complete healing rate for Crohn’s disease (CD) perianal fistulas [16,19,20,22,24,25,26,27,28].

However, 65 patients achieved complete fistula resolution following treatment, resulting in a complete healing rate of 48.05% (95% CI [31.19; 65.11], chi^2^ = 25.18; I^2^ = 68%) (Figure 3).

To attain greater comprehension of PRP injection treatments, subgroup analyses were conducted to compare various application methods, including stromal vascular fraction (SVF)-derived mesenchymal stem cells (MSC) combined with adipose-derived mesenchymal stem cells (ASCs), and local/intra-fistular application of PRP alone.

**Figure 4:**
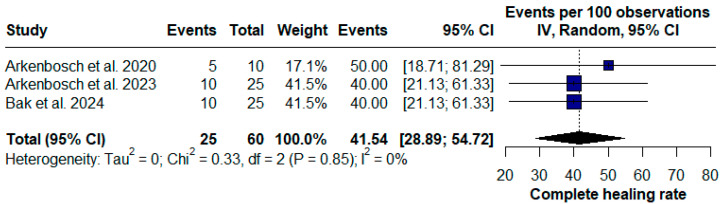
Forest plot of the platelet-rich plasma (PRP) injection subgroup with stromal vascular fraction (SVF) therapy for Crohn’s disease (CD) perianal fistulas [19,20,22].

**Figure 5:**
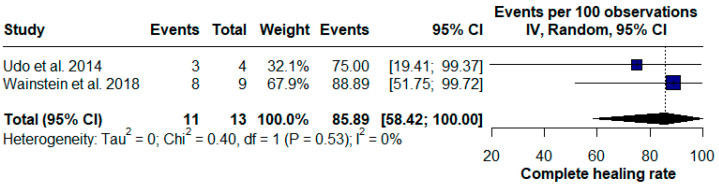
Forest plot of the subgroup of platelet-rich plasma (PRP) injection with adipose-derived mesenchymal stem cells (ASCs-PRP) for Crohn’s disease (CD) perianal fistulas [26,27].

When analyzing all published unfiltered data, the association of PRP injection with the stromal vascular fraction (SVF) presented a complete fistula healing rate of 41.54% [95% CI (28.89; 54.72), Chi^2^ = 0.33; I^2^ = 0%] (Figure 4), while the association with ASCs presented better results, with an efficacy of 85.89% (95%CI [58.42;100.00], chi^2^ = 0.40, I^2^ = 0%) (Figure 5).

**Figure 6:**
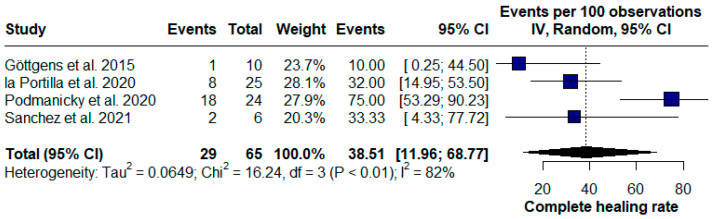
Forest plot of the subgroup platelet-rich plasma (PRP) injection alone for Crohn’s disease (CD) perianal fistulas [16,24,25,28].

Unlike the subgroups that utilized combinations of regenerative therapies, the local application of PRP injections alone demonstrated moderate efficacy but was less effective than the combination therapies, with a lower complete healing rate of 38.51% (95% CI [11.96; 68.77], chi^2^ = 16.24, I^2^ = 82%) (Figure 6).

**Figure 7:**
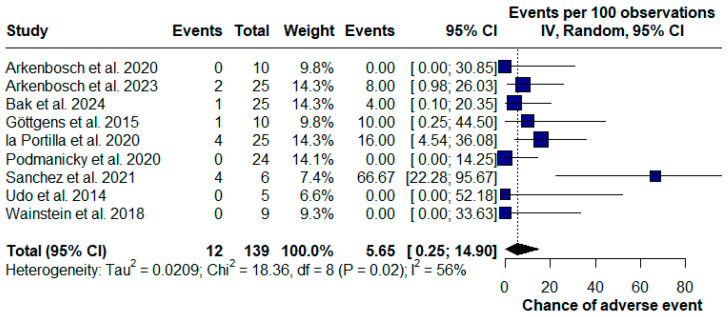
Forest plot of adverse events with PRP treatment for Crohn’s disease (CD) perianal fistulas [16,19,20,22,24,25,26,27,28].

A total of 12 adverse events were reported, including infection, postoperative pain, and minor bleeding at the injection site. However, no severe complications or life-threatening conditions were reported. The adverse event rate for the 12 cases was 5.65% (95% CI 0.25–14.90) (Figure 7).

**Figure 8:**
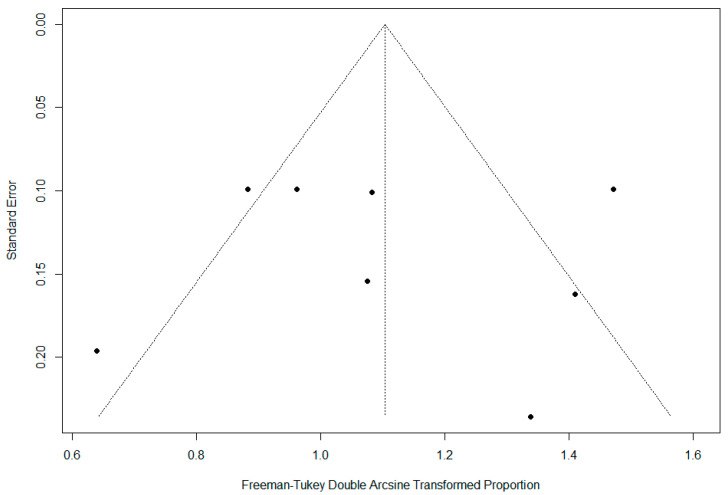
Funnel plot demonstrating publication bias.

A funnel plot and an asymmetry test were performed to address significant heterogeneity. The funnel plot exhibited an asymmetric shape (Figure 8). However, the Egger test results were not significant (*p* = 0.99), suggesting a lower susceptibility to publication bias.

**Figure 9:**
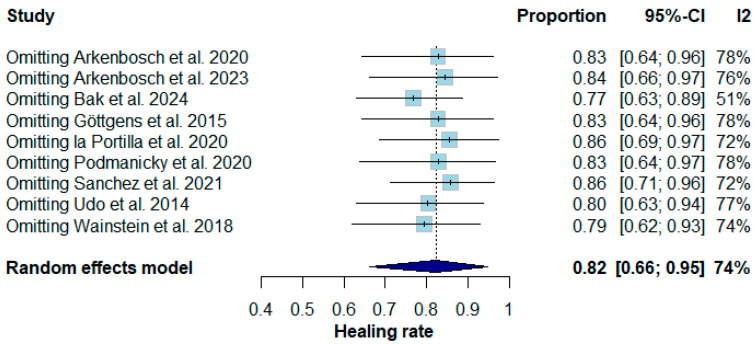
Results of leave-one-out analysis showing the impact of individual data points on complete healing rate [16,19,20,22,24,25,26,27,28].

Because some studies associated PRP injection with other techniques, and others were not fully available, a sensitivity analysis was conducted (Figure 9). This sensitivity analysis showed that, after withdrawing from one study, the summary proportion remained relatively stable, ranging from 0.77 to 0.86. The stability of the summary proportions across the leave-one-out analyses suggests that no single study disproportionately influenced the overall results.

### 2.3. Efficacy of Platelet-Rich Plasma Injections for Treating Colonic CD

Only one study has demonstrated the role of PRP in refractory colonic CD, showing short-term benefits and potential for broader applications. The disease activity was assessed using clinical and endoscopic indexes [23]. Four patients showed decreased endoscopic scores and achieved clinical remission, including the absence of joint pain. No adverse events were observed.

Table 1 summarizes the articles of the systematic review and meta-analysis, including the case series, types of studies, therapeutic methods, techniques used for PRP preparation, and clinical outcomes. The preparation methods for PRP across the studies involved collecting 40–60 mL of peripheral venous blood, followed by centrifugation to separate components into red blood cells, platelet-poor plasma (PPP), and platelet-rich plasma (PRP). In most cases, PRP was concentrated six to eight times higher than baseline levels using specialized systems like GPS-III or SmartPrep^®^. Some protocols included activation with calcium chloride or thrombin-coated syringes to enhance growth factor release. PRP was typically combined with adipose-derived stem cells (ASCs) or stromal vascular fraction (SVF) to create a platelet-rich stroma (PRS), which was injected into fistula tracts and internal openings.

## 3. Discussion

CD is a chronic condition that significantly affects the daily life of patients, causing painful gastrointestinal symptoms and various complications. Fistulas, which affect up to 50% of patients, cause considerable morbidity, including permanent sphincter and perineal tissue destruction, as well as professional and personal disabilities [29].

Depending on the location and severity of CD, treatment options include both clinical and surgical approaches. Immunosuppressants are the first-line treatment for mild CD [30]. Infliximab is recommended for active CD refractory to conventional treatment and is the preferred option for fistulizing CD. The response to anti-TNFα therapy is associated with significant transcriptional regulation, including IL1B, S100A8, and CXCL1, which correlate with endoscopic activity. Notably, patients who did not respond to anti-TNFα therapy exhibited a mixed gene expression signature, with sustained elevated levels of IL1B, IL17A, and S100A8, while also showing significant modulation of other genes commonly upregulated in active CD, such as IL6 and IL23p19 [31].

Despite recent advances in treatment options that have allowed some patients to achieve fistula closure and fibrosis of the fistula tract, identifying effective and comprehensive treatments for CD remains a complex and challenging task. This complexity arises primarily because of the multifactorial nature of the disease, where genetic predispositions, microbiological influences, and immunological dysregulation play pivotal roles in the development and persistence of CD-related fistulas. These factors contribute to the difficulty in achieving long-term remission and necessitate a multifaceted therapeutic approach tailored to the individual patient’s needs [32].

**Figure 10:**
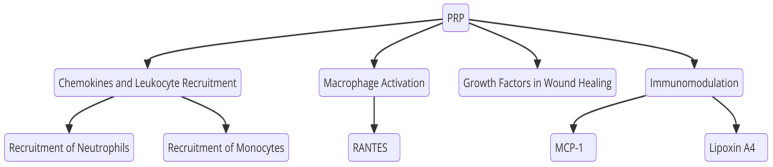
Immunological effects of platelet-rich plasma (PRP). RANTES = Normal T Cell Expressed and Secreted. MCP-1 = monocyte chemotactic protein-1.

Regenerative medicine is an emerging therapeutic approach aimed at the reconstruction of tissues and organs. Incorporating growth factors, anti-inflammatory agents, drugs, and antibiotics can significantly enhance regeneration [9]. Among these novel approaches, PRP has gained attention because of its ability to release multiple growth factors that are crucial for cellular proliferation and angiogenesis. PRP initiates activation of the platelet cascade, resulting in a more abundant reserve of stable fibrinogen than that produced by the polymerization of autologous blood after the addition of exogenous thrombin [33]. Platelet-rich plasma (PRP) has also garnered significant attention in regenerative medicine due to its dual role in promoting tissue repair and modulating immune responses. PRP is rich in growth factors such as Platelet-Derived Growth Factor (PDGF) and Transforming Growth Factor Beta (TGF-β), which support all three phases of wound healing: inflammation, proliferation, and remodeling [34,35]. These growth factors facilitate critical biological processes, including hemostasis, angiogenesis, and extracellular matrix synthesis, all contributing to more efficient wound healing. In addition to its regenerative capabilities, PRP also exerts notable immunomodulatory effects. Key chemokines like Chemokine (C-X-C motif), Ligand 7 (CXCL7), Platelet Factor 4 (PF4), and Regulated upon Activation, Normal T Cell Expressed and Secreted (RANTES), stored in platelets, play pivotal roles in leukocyte recruitment and function [12,36]. CXCL7 attracts and activates neutrophils, while RANTES regulates monocytes and activates T cells, eosinophils, and dendritic cells. PF4 recruits leukocytes and promotes the anti-inflammatory M2 macrophage phenotype, aiding tissue healing [37]. In addition to its role in leukocyte recruitment, PRP significantly influences macrophage activation. PF4, a prominent PRP component, promotes the move of macrophages towards the M2 phenotype, a state associated with anti-inflammatory properties and enhanced tissue healing. This M2 macrophage polarization is crucial for dampening excessive inflammation and facilitating tissue repair [37]. PRP also provides essential growth factors that support the three critical phases of wound healing: inflammation, proliferation, and remodeling. Growth factors such as PDGF (Platelet-Derived Growth Factor) and TGF-β (Transforming Growth Factor Beta) are abundant in PRP. They promote hemostasis, stimulate angiogenesis, and enhance extracellular matrix synthesis, all contributing to more efficient wound healing [38]. PRP suppresses the production of pro-inflammatory markers like the monocyte chemotactic protein-1 (MCP-1) while simultaneously increasing levels of RANTES (Regulated upon Activation, Normal T cell Expressed and Secreted) and Lipoxin A4 (LXA4). LXA4, in particular, plays a key role in limiting inflammation and fostering tissue regeneration, further solidifying PRP’s role in modulating immune responses to support healing processes [39]. Figure 10 illustrates these mechanisms. Additionally, platelet-rich fibrin (PRF) presents an alternative approach, where the structure of the concentrate permits a gradual release of proteolytic growth factors [40].

**Figure 11:**
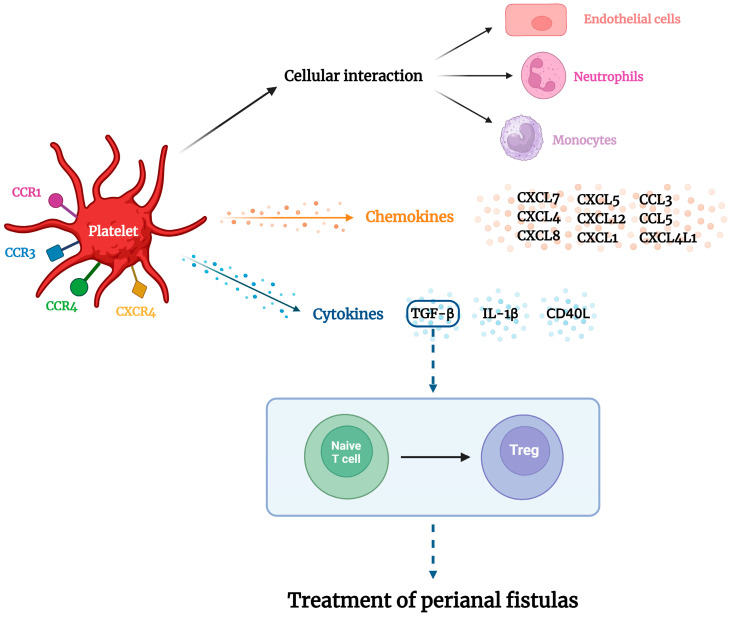
Molecular mechanisms involved in platelet-rich plasma (PRP) therapy may constitute a future perspective for treating Crohn’s disease (CD) perianal fistula.

Most studies included in this systematic review and meta-analysis were observational, small-scale clinical trials. The methodologies range from pilot studies to controlled clinical trials focusing on evaluating the efficacy and safety of regenerative therapies, particularly PRP, ASC, and SVF–PRP, for the treatment of complex perianal fistulas and refractory abdominal CD. The combined effectiveness of achieving complete healing across all studies included in this review was approximately 48.05%. This indicates that less than half of the patients experienced complete fistula closure and healing. In contrast, the partial healing rate was significantly higher, with an effectiveness of approximately 82.44%, indicating that most patients experienced some improvement, although resolution was not complete. PRP therapy has shown sustained healing effects, with most patients maintaining fistula closure for up to one year post-treatment. The underlying mechanisms of action of PRP in the healing of CD perianal fistulas (CDPF) are illustrated in Figure 11. PRP’s multifaceted effects on chemokine signaling, macrophage activation, growth factor release, and immunomodulation position it as a promising therapeutic tool in regenerative medicine and immune regulation [12].

The typical procedure involves the injection of autologous PRP near the internal openings and fistula tracts, often following drainage and closure of the internal openings. Studies investigating the combination of SVF and PRP for the treatment of complex CDPF have shown promising but varied outcomes. Stromal vascular fraction (SVF) contains a heterogeneous mixture of stem cells, growth factors, and immune-modulating components that promote tissue regeneration and reduce inflammation. Combined with PRP, rich in growth factors and cytokines, this therapy enhances the body’s natural healing processes. The PRP component accelerates tissue repair and angiogenesis, while the SVF helps modulate the immune response, preventing chronic inflammation often associated with Crohn’s fistulas [41]. In a cohort of 10 patients, 50% achieved fistula closure within six months, with a notable reduction in Van Assche scores, indicating decreased fistula severity. Patient-reported outcomes varied, with some experiencing significant improvement, while others noted little to no effect, highlighting the need for further research to optimize treatment protocols [19]. A subsequent study included 25 patients, most of whom had previously undergone fistula surgery. This study found complete radiological healing in 9 of 24 patients and a gradual increase in clinical response over 12 months [21]. Although some patients experienced significant healing, the overall success rate was lower than that reported in earlier studies, suggesting the possibility of either more challenging cases or the need for optimized treatment protocols.

The long-term outcomes of the 25 patients included in the follow-up analysis in 2024 revealed that 44% required at least one minor unplanned re-intervention, such as incision and drainage. At follow-up, 88% of the patients achieved complete clinical closure, with 75% showing full radiological closure. Although all patients initially achieved partial clinical closure, 8% experienced recurrence. Importantly, no recurrence was observed in patients who achieved complete MRI closure, and 82% of those with clinical closure achieved full radiological closure [22].

The same research group analyzed the cellular composition of subcutaneous lipoaspirate and platelet-rich stroma (PRS) from 23 patients with CDPF and compared the results with 11 non-inflammatory bowel disease controls. Notably, this study involved the same cohort of patients as previous studies, but with a different approach. Their findings revealed that PRS samples from patients with CD exhibited a higher concentration of cells per milliliter, with a significant increase in myeloid cells, particularly those displaying a regulatory M2/M1 phenotype [20].

Other authors have assessed the effectiveness of SVF and PRP for the treatment of perianal fistulas in a general patient population. In a study involving 40 patients, SVF–PRP injections administered after seton drainage resulted in an 85% clinical healing rate at four months. However, 16% of these patients still had active fistulas detectable on magnetic resonance imaging (MRI) or clinical examination at 12 months. Only five patients had CD; however, the study did not provide detailed outcomes specific to this subgroup [42].

We identified a few studies that explored the use of PRP in combination with different surgical techniques for treating CDPF, which yielded varied results. A smaller clinical study investigated the effectiveness of combining mucosal advancement flap (MAF) surgery with PRP injection. Conducted between 2009 and 2014, the study included 10 patients, half of whom had previously undergone fistula surgery. After seton placement, combination therapy achieved a 70% healing rate at one year, with a 10% recurrence rate and 20% persistence of fistulas. The mean follow-up period was 23.3 months, with one patient developing a postoperative abscess. The median Vaizey score, which indicates continence, was 8.0, suggesting moderate-to-severe impairment [16]. By combining PRP with seton placement, an alternative, less invasive procedure can be performed with lower infection and recurrence rates, leading to improved clinical outcomes and a more rapid recovery. This is especially useful in complex or recurrent fistulas where traditional methods may fail. Additionally, the combination of PRP with other therapies is essential for maintaining anal sphincter function while minimizing complications [16,43].

In a study involving 25 patients with established perianal CD, autologous PRP was prepared in both platelet-rich and -poor fractions for localized intra-fistular injections. The treatment resulted in a complete healing rate of 33.3% at 24 weeks, which had increased to 40% at 48 weeks. Additionally, the number of visible external openings was reduced. However, no significant change was observed in the Perianal Crohn’s Disease Activity Index (PCDAI) [24].

Additionally, a study involving 25 patients with complex CDPF treated with PRP demonstrated a 75% complete healing rate, significantly improving both the Perianal Crohn’s Disease Activity Index (PCDAI) and MRI scores. The treatment was well tolerated, with sustained reductions in PCDAI scores over six months, with these results sustained in nearly all cases at 12 months, reflecting significant and lasting improvement. The Van Assche MRI score notably decreased from 13 to 10. All patients had non-cutting setons for at least six weeks before the study intervention. During the operation, the autologous PRP was prepared by centrifuging 60 mL of peripheral blood using the Harvest SmartPrep© System. After removing the seton, internal openings were closed with a PDS 2/0 single suture, and PRP was injected near the internal openings and along the fistula tracts [25].

Two other studies described the use of adipose-derived stem cells (ASCs) and PRP in combination with a flap advancement technique for treating adult patients diagnosed with CDPF who had complex perianal fistulas refractory to previous surgical and/or biological treatments. The first study involved nine patients (seven women) with a median age of 36 years (range 23–57 years) who were treated for 11 fistula tracts, including two pouch-vaginal fistulas. This study evaluated a small cohort of patients with CDPF treated with endorectal advancement or muscle advancement flaps (MAFs), and for patients with ileal pouches, adipose-derived stem cells (ASCs) and platelet-rich plasma (PRP) yielded satisfactory long-term outcomes. After a median follow-up of 31 months (range 21–37 months), complete healing was achieved in 91% (10/11) of the fistulas, whereas 9% (1/11) showed partial healing. No relapse or adverse reactions were observed at the end of the follow-up period. Significant improvements were also recorded in the Perianal Disease Activity Index (PCDAI) and Inflammatory Bowel Disease Questionnaire scores following the procedure [26].

A two-stage approach was implemented in the second study, which involved five patients for whom anti-TNF therapy and/or surgery had previously failed. This procedure included initial fistula mapping, seton placement, lipoaspiration, and ASC culture, followed by flap advancement and ASC–PRP implantation. At four-month follow-up, complete healing was achieved in three of four patients, with the remaining patient showing partial healing and no radiologic evidence of a persistent fistula. No peri- or postoperative complications related to ASC were observed [27]. When combined with adipose-derived stem cells (ASCs), PRP may enhance the microenvironment by promoting stem cell survival, proliferation, and differentiation, potentially maximizing their regenerative potential and improving tissue repair.

The combination of ASCs and PRP shows excellent potential for treating complex fistulas, particularly in cases where conventional treatments have failed. ASCs can differentiate into cells from multiple germ layers, contributing to tissue repair through paracrine signaling of growth factors. While ASCs offer fusogenic cells that can regenerate new tissue and restore function, PRP enhances wound healing by overexpressing key growth factors that promote cellular growth and reduce inflammation [44]. Although combined therapies may offer superior results for more complex or refractory cases, it remains unclear if they should be universally applied. PRP monotherapy might be sufficient in less severe cases or when a less invasive approach is warranted. Therefore, further research is needed to define which clinical situations would benefit most from PRP monotherapy versus combination therapy, considering factors such as disease severity, fistula complexity, and patient response to previous treatments.

Furthermore, recent evidence suggests that PRGF may be a valuable adjuvant for the treatment of complex fistulas in perianal CD. Plasma rich in growth factors (PRGF) is a regenerative therapy derived from the patient’s blood, containing a high concentration of growth factors promoting tissue repair and healing. It is generally easier to prepare and administer than other treatments and, purely autologous, it carries a relatively lower risk of complications [45,46]. One study demonstrated the ability of PRGF to enhance healing while maintaining anal continence, with a reported recurrence rate of 66.7%. This study found no significant differences in recurrence rates based on age, sex, comorbidities, fistula type or location, medical treatment, or seton use during surgery. The PRGF-poor fraction was used to create a three-dimensional fibrin matrix, which was applied to fill the curetted fistula tract, providing structural support and sustained release of growth factors. No complications were reported, highlighting PRGF’s safety and potential effectiveness [28].

These studies highlight variability in the effectiveness of PRP-based treatments for CDPF. Although PRP shows potential, especially when combined with other surgical techniques, its efficacy as a stand-alone treatment or in combination with simpler procedures is generally low. The use of setons and flap advancements in conjunction with PRP appears to provide better outcomes, although achieving consistent and complete healing remains a challenge across the board. This suggests that although PRP can be an important component of treatment, it may require a combination of more aggressive or varied surgical techniques to optimize patient outcomes in complex cases of PCD.

This systematic review aimed to explore the use of PRP injections in the treatment of CD. Most available research has focused on perianal CD, with only one study addressing PRP therapy for colonic CD. This case series involved five patients with refractory CD who received 12 weekly subcutaneous PRP injections. The results showed significant short-term benefits, including a decrease in endoscopic scores in 80% of patients and partial healing of the colonic mucosa with a reduction in the Crohn’s Disease Endoscopic Index of Severity (CDEIS). Additionally, two patients experienced resolution of joint pain and decreased perianal discharge, and three gained weight. No adverse effects were reported, suggesting that PRP could be a safe and effective therapy for short-term symptom relief in anti-TNFα refractory CD [23].

Although these studies offer valuable insights into the diverse treatment modalities for perianal fistulas in patients with CD, their utility is limited due to several methodological limitations. Small sample sizes, single-center designs, and the absence of comparative effectiveness data underscore the need for extensive multicenter trials to establish the most efficacious interventions. This heterogeneity in preparation techniques and patient populations makes it difficult to generalize the results or draw firm conclusions about efficacy. While PRP shows promise as a safe and adjunctive therapy, more extensive randomized controlled trials will be necessary to confirm its effectiveness and to standardize treatment protocols, thereby improving the reliability of future meta-analyses. Furthermore, contextualizing these findings within the broader landscape of existing evidence, including systematic reviews and meta-analyses, is imperative to garner a comprehensive understanding of the efficacy and safety profiles of these interventions.

## 4. Materials and Methods

### 4.1. Study Design

This systematic review and meta-analysis aimed to evaluate the effectiveness of regenerative therapies, particularly PRP, in the treatment of CD. The review process included defining the research question, identifying databases, determining the search interval, detailing search elements and descriptors, conducting an extensive database search, and applying the inclusion and exclusion criteria.

Studies investigating the use of regenerative therapies, especially PRP, alone or in combination with other treatments, in patients diagnosed with CD were included. This review focused on interventions aimed at regenerating tissues affected by the disease and reported relevant clinical outcomes, such as symptom improvement, lesion healing, and reduction of inflammation. Randomized controlled trials and observational studies published in English, Spanish, French, or Portuguese between 2006 and 2024 were included. The study protocol is registered in PROSPERO (University of York, York, UK) (CRD42024576683).

### 4.2. Research Strategy, Screening, and Data Extraction

The Rayyan QCRI17 system [17] was used to select studies and export the data. Two reviewers independently reviewed the articles, and a third reviewer resolved any conflicts. The search covered databases including PubMed, PubMed PMC, BVS-BIREME, Scopus, Web of Sciences, Embase, Cochrane, EBSCOhost, Proquest, and EndNote. The articles were thoroughly read, and inconsistencies were removed.

The analysis and discussion adhered to the Preferred Reporting Items for Systematic Reviews and Meta-Analyses (PRISMA) guidelines [18].

The keywords “Crohn Disease”, “Inflammatory Bowel Diseases”, and “Platelet-Rich Plasma” were chosen after reviewing related literature. Boolean operators “OR” and “AND” were used to expand and guide the search. The search terms used included Crohn Disease OR Inflammatory Bowel Diseases AND Platelet-Rich Plasma.

The Population, Interventions, Comparators, and Outcomes (PICO) framework has been widely used. The PICO framework was adopted as the study design. Table 1 outlines the primary aspects of interest in this systematic review.

### 4.3. Statistical Analysis

Statistical analysis was performed using R software (version 4.3.2, R Core Team^®^, Vienna, Austria) and a meta package (R Core Team^®^, Vienna, Austria). Pooled proportions were calculated with a 95% confidence interval (95% CI) using a random-effects model with a generic inverse variance method. The Chi-square test and heterogeneity index (I^2^) were used to evaluate heterogeneity between studies. An asymmetry test funnel plot and sensitivity analysis were used for the bias analysis.

## 5. Conclusions

This review indicates that PRP is a promising alternative for the management of complex CD, particularly perianal fistulas. Combining PRP with other regenerative therapies such as SVF and ASCs, as well as surgical techniques, has enhanced healing outcomes, highlighting the potential of these integrative approaches. Additionally, new proposals for the use of PRP, along with advanced and emerging treatment modalities, such as cell therapy, exosome therapy, and tissue engineering for perianal fistulas, further emphasize the potential of these integrative strategies. This therapy demonstrates substantial promise in promoting tissue repair and modulating the inflammatory response, underscoring the evolving landscape of therapeutic strategies and the need for continued exploration of novel approaches. However, additional large-scale randomized controlled trials are required to confirm long-term efficacy, safety, and optimal integration into clinical practice.

## Figures and Tables

**Table 1:** Results of the leave-one-out analysis showing the impact of individual data points on the overall, complete, and partial healing rate of CD perianal fistulas (CDPF).

Study	Research Design	Therapeutic Method (Intervention Techniques and Application Approaches)	PRP Preparation Methods	n = Patients	Overall Healing	Complete Healing	Partial Healing
Arkenbosch et al., 2020 [19]	Prospective observational study	SVF–PRP (local application) Fistula curettage and closure of the internal opening.	The process involves aspirating 15 mL of fat from both sides of the posterior superior iliac spine, followed by centrifugation and mechanical fractionation to produce 1 mL of stromal vascular fraction (SVF). At the same time, 15 mL of blood was drawn and centrifuged to obtain 4–5 mL of PRP with a platelet concentration of 5 × 10^8^/mL. Finally, 1 mL of SVF is combined with 5 mL of PRP to form platelet-rich stroma (PRS) for therapeutic application.	10	8	5	3
Arkenbosch et al., 2023 [20]	Prospective observational study	SVF–PRP (local application) Fistula curettage and closure of the internal opening.	Adipose tissue and venous blood were used to obtain stromal vascular fraction (SVF) and platelet-rich plasma (PRP). The PRS mixture was prepared by combining approximately 1 mL of SVF with 5 mL of PRP.	25	17	10	7
Bak et al., 2024 [22]	Pilot study	PRS (SVF + PRP) (local application) Fistula curettage and closure of internal fistula opening.	SVF was extracted from adipose tissue after fat harvesting and mechanical processing. PRP was obtained by centrifuging whole blood. The PRS mixture, typically consisting of 1 mL of SVF and 5 mL of PRP, was injected into the fistula walls and internal openings to promote healing.	25	25	10	15
Göttgens et al., 2015 [16]	Prospective study	Mucosal advanced flap and PRP (local application).	For PRP preparation, 55 mL of the patient’s blood was collected, achieving a platelet concentration six to eight times higher than baseline levels. During injection into the fistula tract, the PRP was activated with a thrombin-coated syringe. The Gravitational Platelet Separation III (GPS-III) system, developed by Cell Factor Technologies, Biomet, prepared the PRP.	10	8	1	7
La portilla et al., 2020 [24]	Pilot study	PRP (local application of platelet-rich and platelet-poor fractions)	40 mL of peripheral venous blood was collected in a sterile container with 3.8% sodium citrate (Venoject^®^). The blood was centrifuged at 1800 rpm for eight min, separating the upper plasma fraction from the lower fraction containing leukocytes and erythrocytes. The plasma was divided into platelet-poor plasma (PPP) and platelet-rich plasma (PRP). A 10% calcium chloride solution (50 μL per mL of plasma) was added to the PRP, forming a stable fibrin polymer for therapeutic use.	25	15	8	7
Podmanicky et al., 2020 [25]	Prospective, uncontrolled, single-center study	Non-cutting setons prior. Closure of the internal openings and PRP (local application).	60 mL of peripheral blood is drawn from the patient during the procedure. The blood is centrifuged using the SmartPrep^®^ system to efficiently separate its components into three layers: red blood cells at the bottom, platelet-poor plasma (PPP) at the top, and platelet-rich plasma (PRP) in the middle, containing concentrated platelets. The SmartPrep^®^ system enhances platelet yield, producing PRP with a significantly higher platelet concentration than baseline blood, which boosts its regenerative potential. The PRP was prepared in real time during surgery to ensure it was fresh and ready for immediate use.	24	19	18	1
Sanchez et al., 2021 [28]	Retrospective observational	Closure of the internal fistulous orifice and plasma rich in growth factors (PRGF). 50% was injected into the submucosa of the closed IFO, and the other 50% was injected into the fistulous tract.	The blood was centrifuged to separate its components, isolating the platelet-rich plasma (PRP) layer from red and white blood cells. The plasma-rich portion was carefully collected to avoid contamination with inflammatory cells. Calcium chloride was then added to activate the platelets, triggering the release of bioactive growth factors.	6	2	2	0
Udo et al., 2014 [27]	Prospective cohort study	ASC + PRP (local application) Seton placement and closure of internal fistula opening (flap advancement technique).	Adipose tissue was harvested through lipoaspiration and processed to isolate adipose-derived mesenchymal stem cells (MSCs), yielding 100–120 million MSCs for therapeutic use. Blood was drawn from the patient and centrifuged to separate and concentrate platelet-rich plasma (PRP). The MSCs and PRP were combined and injected to enhance tissue regeneration and promote fistula healing.	4	4	3	1
Weinstein et al., 2018 [26]	Single-center, prospective observational pilot study	ASC + PRP (local application) Seton placement and closure of internal fistula opening (flap advancement technique).	For PRP preparation, 40–60 mL of peripheral blood was collected from the patient and centrifuged to isolate platelet-rich plasma (PRP). A mixture of 100–120 million adipose-derived stem cells (ASCs) was combined with the prepared PRP. The ASC–PRP mixture was injected into the internal fistula opening and along the fistula tract. The final portion of the ASC–PRP solution was activated with calcium before filling the fistula tract, forming a biological plug to support healing.	9	9	8	1

CDPF: Crohn’s disease perianal fistula; ASC: adipose-derived stem cells; PRP: platelet-rich plasma; PRS: plasma-rich stroma; SVF: stromal vascular fraction; ASC + PRP: autologous stromal vascular fraction-derived mesenchymal stem cells and PRP; IFO: internal fistulous orifice; PRGF: plasma rich in growth factors.

## Data Availability

Not applicable.
